# Risk-Reducing Salpingo-Oophorectomy and the Use of Hormone Replacement Therapy Below the Age of Natural Menopause

**DOI:** 10.1111/1471-0528.16896

**Published:** 2021-10-20

**Authors:** R Manchanda, F Gaba, V Talaulikar, J Pundir, S Gessler, M Davies, U Menon, on behalf of the Royal College of Obstetricians and Gynaecologists

## 1 Introduction

Ovarian cancer is the commonest cause of death among gynaecological cancers.^1^ Despite advances in drug discovery and treatment strategies, long term survival rates have improved only marginally over the last 30 years, with 10-year survival rates at around 30%. Ovarian cancer screening is unavailable on the NHS. There are screening tools, such as the Risk of Ovarian Cancer Algorithm (ROCA), which have been developed for early diagnosis of ovarian cancer. The ROCA was evaluated in low risk women in the UK Collaborative Trial of Ovarian Cancer Screening (UKCTOCS),^2^ and in high risk women in the UK Familial Ovarian Cancer Screening Study (UKFOCSS).^3^ In both studies a high proportion of women with earlier stage disease were detected. However, long term follow-up data from UKCTOCS did not show a delayed mortality benefit and hence, screening is not currently recommended in general population women.^2,4,5^

In the absence of robust screening tools, preventative surgery is the key strategy to reduce the risk of ovarian cancer. In women at increased risk of ovarian cancer (Appendix I), risk-reducing salpingo-oophorectomy (RRSO) is the most effective method of prevention. Oophorectomy alone is inadequate and clinically inappropriate for prevention. Given the evidence that the majority of high-grade serous cancers arise from a fallopian tube, it is essential that both tubes and ovaries are removed. In *BRCA1/BRCA2* carriers,^4^ RRSO has been found to be effective in significantly reducing ovarian cancer risk and mortality (Appendix I). A 2–4% residual risk of primary peritoneal cancer remains post RRSO in *BRCA1 / BRCA2* carriers,^6^ and only a few cases have been reported in those with Lynch syndrome. While earlier studies suggested premenopausal RRSO halves the risk of breast cancer in *BRCA1/BRCA2* women,^7^ more recent reports showed no such reduction.^8^ RRSO is associated with high satisfaction rates of over 85%, reduced cancer worry and lower perceived cancer risk.^9^ Premenopausal oophorectomy with premature loss of ovarian function is however associated with menopausal symptoms (vasomotor symptoms), poorer sexual function^9,10^ and detrimental impact on bone health.^11,12^ Data from low risk general population women show a negative impact on cardiac^13^ and neurological health from oophorectomy, but corresponding data from high risk women are lacking.^14,15^ These consequences predominantly occur in women who do not take HRT. Potentially lower survival has been reported in low risk women under 50 years of age who underwent premenopausal oophorectomy and did not use HRT.^16,17^ HRT is indicated to relieve symptoms and prevent/minimise any complications and adverse impact on long-term health.

## 2 Indications for RRSO

RRSO has been traditionally offered and shown to be both clinically effective and cost-effective in *BRCA1*/*BRCA2* carriers^18^ and in women with Lynch syndrome (mismatch repair gene [*MLH1, MSH2* or *MSH6*] mutation carriers).^19^ A concomitant hysterectomy is undertaken in those with Lynch syndrome as they also have a 40–60% lifetime risk of endometrial cancer.^20^ In the UK, given the historic restricted access to genetic testing, RRSO has been offered to women from high risk families with an estimated 10% or more lifetime ovarian cancer risk who were unable to access gene testing.^21^ However, there has been significant variation in the family history based criteria used, with some identifying women in the intermediate risk category (around 7–10%) for RRSO.

RRSO has been shown to be cost-effective at lifetime ovarian cancer risk thresholds of more than 4–5%.^22,23^ RRSO can therefore also be offered to women with moderate risk gene mutations including *RAD51C, RAD51D*, and *BRIP1* (5–13% lifetime ovarian cancer risk),^24–26^ as well as selected women with a significant family history of ovarian cancer (e.g. one or two first-degree relatives with ovarian cancer)^27,28^ who are at intermediate risk (5-10% lifetime risk).^29,30^ *PALB2* has been confirmed as a moderate risk ovarian cancer gene, with some now supporting RRSO in these women, while others cite the limited evidence for this. RRSO can be considered for women with *PALB2* mutations following a non-directive counselling process taking into account additional risk and protective factors, and preferably carried out near/after menopause (see Appendix I for details). Family history should be incorporated into the individualised risk assessment process for all women. In cases where ovarian cancer risk assessment appears complex or difficult, it is important to seek advice from a specialist with greater expertise, such as a clinical geneticist or gynaecologist/gynaecological oncologist with special interest in genetic risk assessment or hereditary cancer risk management.

## 3 Timing of RRSO

RRSO decision making is a complex process, and timing needs to be individualised following informed counselling of the pros and cons (Appendices II and V), taking into account clinical factors and personal preference. RRSO is usually offered once a family is complete. There are occasional exceptions when women undergo IVF and have embryos stored prior to RRSO in order to complete their family later. In women with early onset cancers in the family it may also be undertaken from up to 5 years before the earliest recorded age of onset of ovarian cancer in the family. It is typically offered from 35–40 years for *BRCA1* carriers, 40–45 years for *BRCA2* carriers, 40–50 years for *RAD51C/RAD51D* carriers, and nearer/after menopause (aged above 45–50 years) for *PALB2* carriers. In *BRIP1* carriers and mutation-negative, intermediate risk women (5–10% lifetime ovarian cancer risk) with a strong family history, it may be delayed until 45–50 years (Appendix I).^29,30^ A significant number of women undergoing RRSO will end up with premature iatrogenic menopause (with the average age of natural menopause being 51 years) requiring HRT. Clearly the issue of risk and age of surgery needs to be individualised and there must be informed discussion with women regarding the consequences of iatrogenic surgical menopause, benefits of HRT, and its risks and limitations so they can make informed decisions (Appendix II). Women are best cared for in dedicated high risk clinics or by multidisciplinary teams involving gynaecologists/gynaecological oncologists with specific interest in care of women at high risk, a psychologist, and clinical nurse and menopause specialists. There should also be links to clinical genetics, breast and colorectal teams.

## 4 The role of hysterectomy

Routine concomitant hysterectomy is justified only in women with Lynch syndrome because of an increased risk of endometrial cancer.^20^ It may be appropriate in a small number of other women for independent gynaecological indications, such as fibroids and adenomyosis.

Few studies have reported an increased risk of serous (subtype) endometrial cancer in *BRCA1* carriers.^31,32^ This comprises a small proportion (approximately 7%) of endometrial cancers,^33^ with the overall population-based lifetime risk for endometrial cancers being 2.4% in the UK and 2.9% in the USA. Moreover, the number of reported serous endometrial cancer cases are small, confidence intervals wide, and the absolute lifetime risk is low (around 3%), and total endometrial cancer risk is not increased in *BRCA1* carriers. Endometrial cancer risk is not increased in *BRCA2* carriers. Therefore, more corroborating data and precision around endometrial cancer risk are needed before hysterectomy in *BRCA1* or *BRCA2* carriers can be routinely advocated.

## 5 Impact of surgical menopause and benefits of HRT after RRSO

Iatrogenic menopause owing to RRSO can be associated with vasomotor symptoms, mood changes, sleep disturbance, reduced libido, vaginal dryness, dyspareunia (painful intercourse) and poorer sexual functioning compared with women who retain their ovaries.^9^ HRT use ameliorates all these symptoms. Despite HRT, the reported symptoms, particularly for sexual dysfunction, remain above those who have not undergone premenopausal oophorectomy.^10^ Specifically, sexual dysfunction following RRSO is reported in up to 74% of women compared with general population levels of 40–45%.^34^

Studies in the general population have reported premenopausal oophorectomy (before natural menopause) is associated with an increased risk of heart disease,^13^ and up to 3% absolute increase in mortality from heart disease in low risk women who have had early surgical menopause and did not take HRT.^13^ An increased risk of stroke has also been reported in low risk women,^13,35^ however, these data were not statistically significant. Other reported potential negative consequences in low risk women include increased incidence of neurocognitive impairment, dementia and parkinsonism.^14,17^ Detrimental consequences have predominantly occurred in women who do not take HRT. Adequate comparable data on cardiac and neurological consequences are lacking for high risk women.^15^ RRSO is associated with elevated bone turnover markers, an increased risk of osteopenia and osteoporosis,^12^ however, data on excess fracture risk are limited.^36^ The impact of estrogen deficiency is related to the duration of lack of estrogen and therefore earlier age at RRSO carries greater risk; this should be a factor in decision making (Appendix II).

HRT is indicated for symptom relief and to ameliorate the adverse long-term consequences of premature menopause following RRSO.^10^ There is evidence that HRT reduces the detrimental impact on bone health (osteoporosis)^37^ and significantly improves quality-of-life^38^ in high risk women.^15,39^ In low risk women it has been found to reduce ischaemic heart disease and associated cardiovascular disease mortality,^13^ and neurological consequences following oophorectomy. A summary of benefits and risks is given in Appendix III. Overall, data in high risk women are limited to short- and medium-term outcomes. Further well-designed studies with long-term outcomes of RRSO and HRT use in high risk women are needed.

### 5.1 Initiation and duration of HRT

In women without previous history of breast cancer, and in the absence of other contraindications, HRT can be offered after counselling to women at increased ovarian cancer risk undergoing early surgical menopause (including *BRCA* carriers) (Appendix II). HRT is commenced immediately postoperatively and is recommended until the mean age of natural menopause (i.e. 51 years)^40^ provided there are no other contraindications.^15,39^ Thereafter, continuation, while not routinely recommended for those at high risk of breast cancer, should only be undertaken based on informed discussion regarding the risks and benefits of taking HRT after the age of natural menopause, taking into account individual circumstances and medical history.

### 5.2 Types of HRT

Estrogen-only HRT (E-HRT) should be used in women undergoing hysterectomy in addition to RRSO. For those with an intact uterus, estrogen is combined with a progestogen (E+P-HRT) to protect against endometrial hyperplasia/cancer. Progestogens can be given cyclically to induce regular withdrawal bleeds, or continuously in a bleed-free formulation. Several systemic HRT preparations are available with different combinations, strengths and routes of administration. In some women additional topical estrogen may be required to treat urogenital atrophy.^41^

Estrogens can be delivered orally or transdermally (subcutaneous implants are no longer distributed in the UK). Transdermal estrogens have a lower risk of venous thromboembolism (VTE), stroke and myocardial infarction than oral preparations.^42^ Vaginal estrogen is not associated with an increased risk of endometrial hyperplasia.^41,43^

Progestogens can be delivered orally, transdermally, or directly in the uterus (progestogen-releasing intrauterine system). The latter is associated with fewer adverse effects than systemic progestogen (Appendix IV).^44^ Oral micronised progesterone may have a better risk profile than synthetic progestogens.^43^

Tibolone is a synthetic steroid with estrogenic, progestogenic and androgenic activity. It can be used as continuous combined HRT to treat vasomotor, psychological and libido symptoms following surgical menopause, while conserving bone mass and reducing the risk of vertebral fractures.^45^

### 5.3 Androgen therapy

Premenopausal oophorectomy reduces free androgen index levels by 50%. Testosterone replacement may benefit women experiencing low energy levels and reduced libido despite adequate estrogen replacement.^46^ Transdermal testosterone improves sexual activity, orgasms, desire, and positively impacts Personal Distress Scale scores in women affected by hypoactive sexual dysfunction following natural/surgical premature menopause, irrespective of E+P-HRT.^47^ Short-term data confirm safety of transdermal testosterone, although some androgenic adverse effects (acne and hair growth) are reported.^47^ However, data specific to high risk women are lacking and impact on breast cancer risk is unknown. There are no licensed preparations for women in the UK, so treatment should be in specialist care settings, with access to hormone assays and monitoring of adverse effects. Off-license preparations of testosterone include gels and subcutaneous implants; use should be evaluated after 3–6 months and usually limited to 24 months.^48^

### 5.4 Adverse effects of HRT

Adverse effects are listed in Appendix IV. These may ameliorate over time, or by changing the type, route of administration or dose of HRT. Persistent irregular vaginal bleeding after 6 months requires investigation.

### 5.5 HRT and breast cancer

A number of observational studies have evaluated HRT use in *BRCA1/BRCA2* carriers after premenopausal RRSO. The mean duration of use reported varies from 3.6–7.6 years (range 0.6–24.4 years in the largest study). Short-term HRT following RRSO in *BRCA1*/*BRCA2* carriers has not been shown to increase breast cancer risk or negate any potential protective effect on subsequent breast cancer risk (Appendix V).^15,38,49–54^ Hence, HRT up to 51 years of age is recommended post RRSO in the absence of any contraindication.^40^ In low risk general population women, E+P-HRT^43^ is associated with increased breast cancer risk, with a meta-analysis suggesting risks may also be increased with E-HRT, although risk levels are much lower than E+P-HRT.^55^ Limited data in *BRCA* carriers have not shown a significant difference in breast cancer risk with E-alone or E+P preparations (compared to non-users), but additional long-term data and larger well-designed studies addressing this issue are needed to corroborate this.^49^ In low risk women E-HRT has a better risk profile than E+P-HRT. More data in high risk *BRCA* women are needed. Although specific data on natural progesterone are lacking in *BRCA1/BRCA2* high risk women, a favourable risk profile is reported in low risk general population women.^56^ Safety data to continue HRT beyond the age of 51 years in high risk women are lacking and its use is not currently routinely recommended. Any decision to continue HRT should be based on a clinical discussion of pros and cons involving the woman and a menopause specialist or gynaecologist experienced in caring for high risk women. However, some women at increased risk of ovarian cancer may not be at increased risk of breast cancer, such as *BRIP1* carriers or women with Lynch syndrome. HRT use in women over 5I years of age may be governed by the same principles as women at population-based risk.

For women with a personal history of breast cancer, HRT is usually contraindicated because of estrogen receptor-positive status. About 24–30% of *BRCA1*-associated breast cancers and 65–79% of *BRCA2*-associated breast cancers are estrogen receptor-positive.^57^ In women with triple-negative breast cancer, HRT can be considered for short-term use on an individual basis, particularly in those with good prognosis. It can also be considered in long term survivors who have undergone bilateral mastectomy, as may happen in some *BRCA* carriers who develop breast cancer. Any decision about HRT use should be multidiscliplinary involving the woman, a breast oncologist and a menopause specialist or gynaecologist experienced in caring for high risk women. For breast cancer patients with vaginal/urogenital symptomatology alone, non-hormonal approaches, such as lubricants and moisturisers, are the first line options. Ospemifene, a newer selective estrogen receptor modulator with an estrogen-like effect in the vagina may potentially be beneficial for symptomatic vulvar and vaginal atrophy (VVA). However, adequate data in women with breast cancer are lacking, with use in one small study^58^ restricted to women with a history of breast cancer 10 years and more prior to enrolment. Consequently, it is not recommended for use in this group of women presently. Intravaginal administration of dehydroepiandrosterone (DHEA) has also been shown to be clinically effective for the symptoms of VVA; however, its use is not yet recommended in women with past history of breast cancer because of insufficient safety data. If non-hormonal options are not effective and symptoms are debilitating, short-term topical estrogen at the lowest effective vaginal dose may be considered following specialist advice (including for estrogen receptorpositive breast cancer with a good prognosis).^59,60^ Professional bodies have suggested that vaginal estrogen should be given with tamoxifen and not aromatase inhibitors.^53,61,62^ The effect of any systemic estrogen absorption may be counteracted by tamoxifen’s mode of action at the receptor level in breast tissue. The evidence base for this is limited. If switching adjuvant therapy is considered, this should involve the breast oncologist with a menopause specialist to consider potential differences in breast cancer recurrence rates as well as symptom control. HRT should be used/prescribed following clinical discussion with the woman, so they are clear about the recommendations and circumstances under which use of HRT is or is not possible.

### 5.6 Other risks associated with use of HRT

#### 5.6.1 Endometrial cancer

Although overall risk of endometrial cancer is not increased post RRSO, specific data on endometrial cancer risk with HRT use in *BRCA* carriers or women at high risk of ovarian cancer are lacking. However, good quality data are available from low risk women.^63^ Consistent with advice for those at low risk, only combined regimens should be used in women with a uterus. In healthy postmenopausal women, continuous combined HRT is associated with a slightly lower risk of endometrial hyperplasia/carcinoma than cyclical regimens.^43^

#### 5.6.2 Venous thromboembolism and stroke

Oral HRT is associated with increased VTE risk, especially during the first year of treatment, and appears to be higher with E+P-HRT than E-HRT. The VTE risk with standard therapeutic doses of transdermal HRT is similar to baseline population risk.^64^ Transdermal HRT should be considered instead of oral preparations for women at increased risk of VTE, including those with a body mass index over 30 kg/m^2^. Women may be commenced on transdermal HRT immediately postoperatively and do not require anticoagulation unless there are additional risk factors for VTE. In low risk women with premature ovarian insufficiency, the absolute risk of stroke is low,^43^ and nor is it significantly increased following surgical menopause.^13,35^ Data specific to high risk women undergoing RRSO are lacking.

### 5.7 Contraindications to HRT after RRSO

There are few contraindications aside from history of breast cancer and personal history of VTE/thrombophilia. However, the latter can be considered for transdermal HRT after discussion of the benefits versus risks and input from haematology specialists on a case-by-case basis. HRT should not be offered if there is undiagnosed abnormal vaginal bleeding, suspected or active endometrial cancer.

### 5.8 Monitoring HRT

After starting HRT, it is advisable to review therapy after 3 months and annually thereafter. While routine tests may not be necessary, investigations should be prompted by specific symptoms or concerns, for example unexpected bleeding. Serum hormone levels are generally not helpful in making treatment decisions. It is important to evaluate and advise on cardiovascular risk factors. Assessment of osteoporosis risk should be carried out. Dual energy X-ray absorptiometry (DEXA) scanning for bone mineral density (BMD) should be considered 1–2 years after RRSO, especially if there are additional risk factors for poor bone health. If BMD is normal and HRT has been prescribed, the value of a repeat DEXA scan is low.^43^ Women with known osteoporosis, a strong family history, or those at increased risk due to the use of aromatase inhibitors for breast cancer should have initial and periodic (every 2–5 years) DEXA scans.^65^ It is not necessary to routinely monitor endometrial thickness while using topical or systemic HRT.

Maintaining HRT compliance is necessary to minimise the detrimental consequences of premature menopause. Poor compliance rates varying from 25–60% have been reported following RRSO in *BRCA* carriers in some studies,^12,49^ with higher uptake rates of approximately 74% reported in women cared for in specialist centres.^66^ Good communication with the general practitioner, and informing women regarding the benefits and risks of HRT is essential to help to maintain compliance.

## 6 Alternatives to HRT

Women with contraindications to HRT and those who decline HRT may consider alternative pharmacological, non-pharmacological and complementary treatments for symptoms of menopause. However, overall evidence for such treatments is limited and they do not address long-term health risks after RRSO.

Three RCTs have demonstrated that cognitive behavioural therapy (CBT) is helpful after natural menopause^67^ and following treatment for breast cancer.^68,69^ Vasomotor symptoms were rendered more tolerable and less intrusive. Both CBT and exercise were effective in diminishing endocrine and urinary symptoms, but only CBT reduced the burden of hot flushes and night sweats, and led to an increase in sexual activity.^68^ CBT may also alleviate low mood or anxiety associated with surgical menopause.^66^ CBT delivered as group therapy^70^ or self-administered are equally effective,^69^ with data supporting an internet-based approach.^71^ While specific trials in RRSO populations are absent, the parallel with cancer-induced menopause makes it reasonable to apply this modality to surgically-induced menopause on clinical grounds and symptom similarity.

Although RRSO-specific data are limited, psychosexual interventions post gynaecological cancer have been effective using CBT, psychoeducation and mindfulness. A small study of similarly structured interventions in 39 women following RRSO showed significant improvements in sexual desire, arousal and satisfaction.^72^

Most pharmacological trials are small studies of short duration. Pharmacological options include selective serotonin reuptake inhibitors (SSRIs), selective noradrenaline reuptake inhibitors (SNRIs), clonidine, gabapentin and betablockers. There is little evidence regarding efficacy and safety of these medications for treatment of menopausal symptoms in young women with surgically-induced menopause. Overall, studies have demonstrated that venlafaxine 37.5 mg titrated up to 150 mg/day, paroxetine 10 mg/day or citalopram 10-30 mg/day are the most effective agents. Clonidine 100 micrograms/day provided significant reduction in the numbers of hot flushes and improved quality-of-life compared with placebo in women with breast cancer, but may have unacceptable adverse effects.^73^

Vaginal lubricants and moisturisers can relieve vaginal dryness during intercourse but do not have systemic effects.^74^ Some evidence suggests phytoestrogens (e.g. isoflavones, black cohosh) may relieve vasomotor symptoms, but data on safety and survival benefits in breast cancer patients are inconsistent. Phytoestrogens are not recommended for breast cancer survivors.

## 7 Lifestyle advice

To address the risk of bone demineralisation and improve cardiovascular health following RRSO, women are advised to maintain a healthy lifestyle, undertake weight-bearing exercise, avoid smoking and excessive alcohol intake, and maintain normal body weight (corresponding to a body mass index 18.5–24.9 kg/m^2^). Exercise may achieve clinically important preservation of bone health among premenopausal women with early breast cancer.^75,76^ Dietary calcium and vitamin D3 supplementation may be required, particularly in women with inadequate vitamin D status and/or calcium intake. Supplementation to achieve a total intake of 1200 mg/day of calcium and 600-1000 IU/day of vitamin D3 has been recommended.^60^ Bisphosphonates are effective in treating osteoporosis, but should only be considered with advice from an osteoporosis specialist.^65^

Women who are more active have fewer menopausal symptoms.^77^ Symptomatic women are advised to undertake regular aerobic exercise, such as swimming or running (the latter being weight bearing has the added benefit of improving bone mineralisation),^77^ lose weight if applicable, and ensure adequate sleep to improve subjective cognitive symptoms. Other general lifestyle advice includes wearing lighter clothing, sleeping in a cooler room, and avoiding possible symptom triggers such as spicy foods, caffeine, smoking and alcohol.^78^

## 8 Opinion

- In the UK, RRSO has previously been offered to women with a high estimated lifetime risk (10% or more) of ovarian cancer. RRSO is the most effective method of preventing ovarian cancer, and is cost-effective in women at 4–5% or greater lifetime ovarian cancer risk. With increasing genetic testing, identification of moderate risk gene mutations, and ability to estimate risk based on family history and other risk factors, there is now an emerging and expanding role for RRSO in women at intermediate risk (5–10% lifetime risk) of ovarian cancer.
- With increasing uptake of RRSO for prevention of ovarian cancer, more women will be exposed to the longterm consequences of premature surgical menopause.
- If not contraindicated, it is important following premenopausal oophorectomy that HRT is offered until the age of natural menopause.
- It is essential that women receive evidence-based information and multidisciplinary input, with advice on HRT, symptom management, specialist counselling and sustained support to deal with various physical, emotional and long-term health consequences.
- Family history should be incorporated into the individualised risk assessment process for all women.
- In cases where ovarian cancer risk assessment appears complex or difficult, it is important to seek advice from a specialist such as a clinical geneticist or gynaecologist/gynaecological oncologist with expertise in genetic risk assessment or hereditary cancer risk management.
- Further research is required to guide the most appropriate form of HRT in high risk young women.

## Supplementary Material

Appendix

## Plain language summary

This paper deals with the use of hormone replacement therapy (HRT) after the removal of fallopian tubes and ovaries to prevent ovarian cancer in premenopausal high risk women. Some women have an alteration in their genetic code, which makes them more likely to develop ovarian cancer. Two well-known genes which can carry an alteration are the *BRCA1* and *BRCA2* genes. Examples of other genes associated with an increased risk of ovarian cancer include *RAD51C, RAD51D, BRIP1*, *PALB2* and Lynch syndrome genes. Women with a strong family history of ovarian cancer and/or breast cancer, may also be at increased risk of developing ovarian cancer. Women at increased risk can choose to have an operation to remove the fallopian tubes and ovaries, which is the most effective way to prevent ovarian cancer. This is done after a woman has completed her family. However, removal of ovaries causes early menopause and leads to hot flushes, sweats, mood changes and bone thinning. It can also cause memory problems and increases the risk of heart disease. It may reduce libido or impair sexual function. Guidance on how to care for women following preventative surgery who are experiencing early menopause is needed.

HRT is usually advisable for women up to 51 years of age (average age of menopause for women in the UK) who are undergoing early menopause and have not had breast cancer, to minimise the health risks linked to early menopause. For women with a womb, HRT should include estrogen coupled with progestogen to protect against thickening of the lining of the womb (called endometrial hyperplasia). For women without a womb, only estrogen is given. Research suggests that, unlike in older women, HRT for women in early menopause does not increase breast cancer risk, including in those who are *BRCA1* and *BRCA2* carriers and have preventative surgery.

For women with a history of receptor-negative breast cancer, the gynaecologist will liaise with an oncology doctor on a case-by-case basis to help to decide if HRT is safe to use. Women with a history of estrogen receptor-positive breast cancer are not normally offered HRT. A range of other therapies can be used if a woman is unable to take HRT. These include behavioural therapy and non-hormonal medicines. However, these are less effective than HRT. Regular exercise, healthy lifestyle and avoiding symptom triggers are also advised. Whether to undergo surgery to reduce risk or not and its timing can be a complex decision-making process. Women need to be carefully counselled on the pros and cons of both preventative surgery and HRT use so they can make informed decisions and choices.
