# Supplementary material for: Risk-Reducing Salpingo-Oophorectomy and the Use of Hormone Replacement Therapy Below the Age of Natural Menopause
Source: BJOG. Author manuscript; Available in PMC 2023 Jul 17. (PMC7614764; doi:10.1111/1471-0528.16896)
Supplement: Appendix [file EMS177339-supplement-Appendix.pdf]

Appendix I: Ovarian cancer risk and risk reduction from RRSO

**Criteria: Mutation based**	**Breast cancer risk (95% CI)**	**Ovarian cancer risk (95% CI)**	**Age for RRSO**[a]
BRCA1[4]	72% (65–79%)	44% (36–53%)	From 35–40 years[b]
BRCA2[4]	69% (61–77%)	17% (11–25%)	From 40–45 years[c]
RAD51C[24]	21% (15–29%)	11% (6–21%)	From 40–50 years[d]
RAD51D[24]	20% (14–28%)	13% (7–23%)	From 40–50 years[d]
BRIP1[25]	No increase	5.8% (3.6–9.1%)	> 45–50 years[e]
PALB2[26g]	53% (44–63%)	~5% (2–10%)	> 45–50 years[d]
	**Endometrial cancer risk (95% CI)**	**Ovarian cancer risk (95% CI)**	**Age for hysterectomy and RRSO**[a]
MLH1[79–81]	37% (30.1–46.5%)	11% (7.4–19.7%)	From 35–40 years
MSH2[79–81]	48.9% (40.2–60.7%)	17.4% (11.8–31.2%)	From 35–40 years
MSH6[79–81]	41.1% (28.6–61.5%)	10.8% (3.7–38.6%)	From 35–40 years
**Criteria: FH based and BRCA status unknown**[h]	**Ovarian cancer familial relative risk**	**Ovarian cancer risk**	
One FDR with OC[27]	~3 (2.4, 3.7)	~5.8% (4.7%, 7.2%)	RRSO may be delayed until 50 years of age (can be influenced by ages and distribution of OC in the family)
Two OC case families[28]	~4 (1.1, 10.4)	~7.7% (2.2%, 18.9%)	
Three or more OC case families[28]	~7.45 (2.0, 19.1)	~13.9% (3.9%, 31.9%)	
**Criteria: FH based and *BRCA*-negative**[h]			
One FDR with OC <50 years[27]	~3.83 (2.4, 6.1)	~7.4% (4.7%, 11.6%)	
One FDR with serous OC[27]	~2.56 (1.8, 3.7)	~5% (3.6%, 7.2%)	
Two OC familial cases[28]	~3–4 (estimated)	~5.8–7.7%	
Three or more OC familial cases[28]	~7 (estimated)	~13%	
Familial high risk BC only[82,83]	≤ 1	Likely population level OC risk (~2%)	RRSO not recommended
**Cancer risk reduction with RRSO**	**Breast cancer risk reduction**	**Ovarian cancer risk reduction**	**Mortality reduction**[f]
BRCA1, BRCA2	Earlier studies: 50% reduction in primary BC risk[7]<br>More recent studies[8]: No reduction in primary BC risk<br>Reduction in premenopausal BC risk in *BRCA2*	80–96% OC risk reduction[6]<br>2–4% residual PPC risk in *BRCA* carriers[6]<br>PPC post preventive surgery in Lynch syndrome is rare	60–77% reduction in all cause mortality[6,84]<br>79% reduction in OC specific mortality<br>56% reduction in BC mortality

 © 2021 Royal College of Obstetricians and Gynaecologists

14710528, 2022, 1, Downloaded from https://obgyn.onlinelibrary.wiley.com/doi/10.1111/1471-0528.16896 by University College London UCL Library Services, Wiley Online Library on [14/06/2023]. See the Terms and Conditions (https://onlinelibrary.wiley.com/terms-and-conditions) on Wiley Online Library for rules of use; OA articles are governed by the applicable Creative Commons License

Appendix I. (Continued)

	No reduction in contralateral BC risk	
Low risk women		94% reduction in OC risk[13]

BC, breast cancer; FDR, first degree relative; FH, family history; OC, ovarian cancer; PPC, primary peritoneal cancer; RRSO, risk-reducing salpingo-oophorectomy.

[a]RRSO may be offered from up to 5 years before the earliest onset OC in the family in women with early onset ovarian cancer.

[b]OC risk in *BRCA1* begins to rise from 35 years of age and increases significantly after 40 years of age.

[c]OC risk in *BRCA2* begins to rise from 40 years of age and increases significantly after 45 years of age.

[d]Although data are limited, OC has not yet been reported in *RAD51C, RAD51D* and *PALB2* carriers under 40 years of age.

[e]OC has not been reported in *BRIP1* carriers under 45 years of age.

[f]Mortality data are based on medium term outcomes with median follow-up time in studies of 3.6–4.3 years[80] and 5.6 years.[5]

[g]*PALB2* was recently confirmed as a moderate risk OC gene, with some now supporting RRSO in these women, while others citing limited evidence for this. RRSO can be considered for women with *PALB2* mutations taking into account additional risk and protective factors, and is preferably carried out nearer/after menopause.

[h]In cases where ovarian cancer risk assessment appears complex or difficult, it is important to seek advice from a specialist with greater expertise such as a clinical geneticist or gynaecologist/gynaecological oncologist with special interest in genetic risk assessment or hereditary cancer risk management.

 © 2021 Royal College of Obstetricians and Gynaecologists

14710528, 2022, 1, Downloaded from https://obgyn.onlinelibrary.wiley.com/doi/10.1111/1471-0528.16896 by University College London UCL Library Services, Wiley Online Library on [14/06/2023]. See the Terms and Conditions (https://onlinelibrary.wiley.com/terms-and-conditions) on Wiley Online Library for rules of use; OA articles are governed by the applicable Creative Commons License

Appendix II: Flowchart for risk-reducing surgery and HRT management

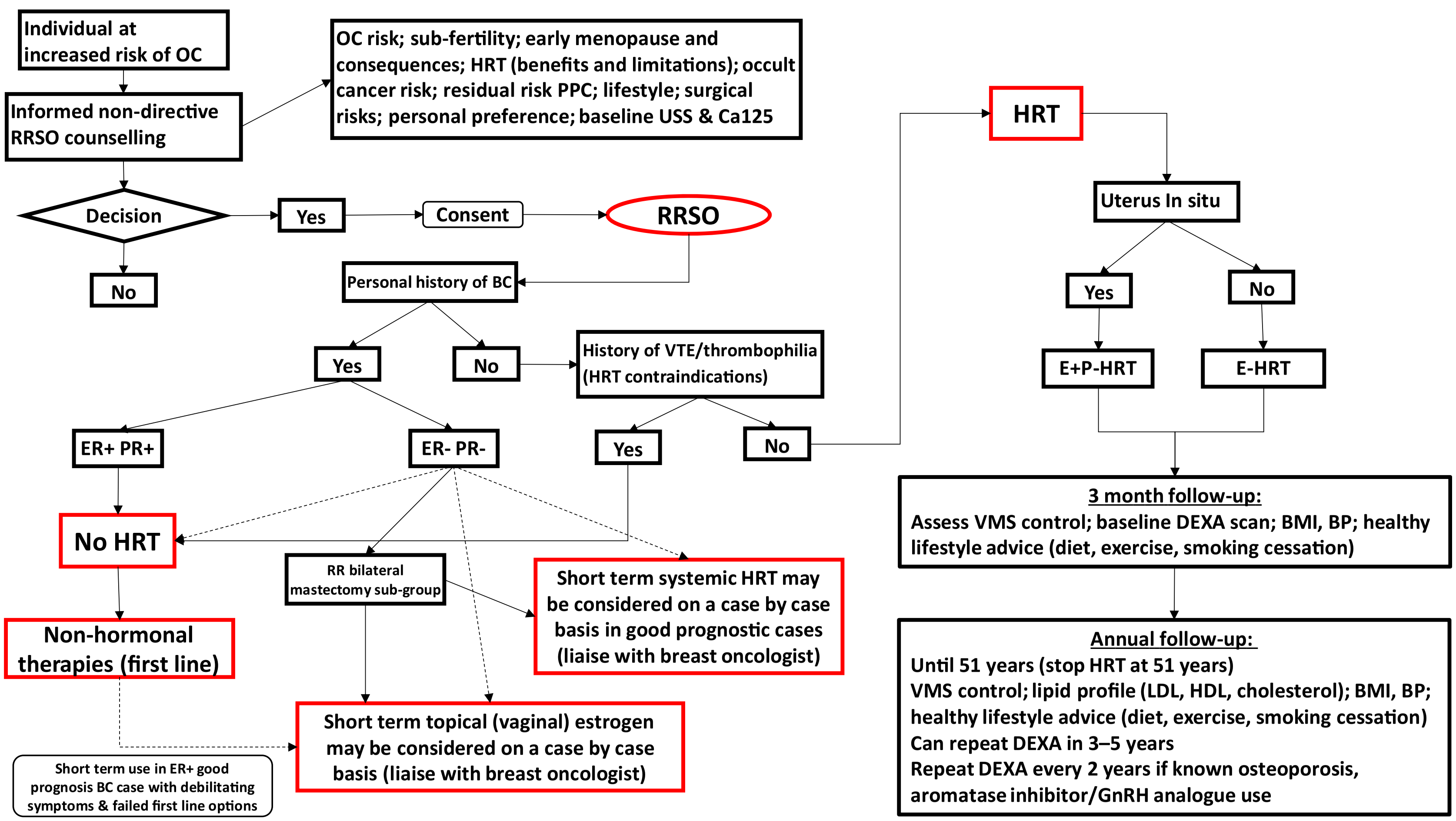

b/l, bilateral; BC, breast cancer; BMI, body–mass index; BP, blood pressure; DEXA, dual-energy x-ray absorptiometry; E, estrogen; ER+, estrogen receptor-positive; GnRH, gonadotrophin-releasing hormone; HDL, high-density lipoprotein; HRT, hormone replacement therapy; LDL, low-density lipoprotein; OC, ovarian cancer; P, progestogen; PPC, primary peritoneal cancer; PR+, progesterone receptor-positive; RR, risk reducing; USS, ultrasound scan; VMS, vasomotor symptoms; VTE, venous thromboembolism.

 © 2021 Royal College of Obstetricians and Gynaecologists

14710528, 2022, 1, Downloaded from https://obgyn.onlinelibrary.wiley.com/doi/10.1111/1471-0528.16896 by University College London UCL Library Services, Wiley Online Library on [14/06/2023]. See the Terms and Conditions (https://onlinelibrary.wiley.com/terms-and-conditions) on Wiley Online Library for rules of use; OA articles are governed by the applicable Creative Commons License

Appendix III: Summary of the benefits and risks of premenopausal RRSO in women at increased risk of ovarian cancer

Impact of premenopausal RRSO: summary of benefits and risks	
**Benefits**	**Comment**
Reduction in OC risk	See Appendix I
Reduction in all-cause mortality	See Appendix I
Reduction in OC specific mortality	See Appendix I
Reduction in BC specific mortality	See Appendix I
Reduction in anxiety and depression	
Reduction in OC worry	
Identification of occult in situ/invasive cancer at histology	5% risk in *BRCA* carriers. Improved survival with identification of early stage disease
**Risks (high risk women)**	**Comment**
Infertility	
Premature menopause	
Vasomotor symptoms	Minimised by HRT
Sexual dysfunction	Improved by HRT, but sexual discomfort remains higher compared to women who retain their ovaries
QoL	No difference in generic QoL with RRSO
Osteoporosis	HRT preserves bone mineral density. No increase in fracture risk reported with RRSO
Primary peritoneal cancer residual risk	2–4% in *BRCA* carriers, rare in Lynch syndrome
Surgical complications	3–4% risk
**Additional risks from oophorectomy in low risk women (with lack of adequate data specific to high risk women)**	**Comment**
Coronary heart disease[a]	Seen predominantly in women who do not take HRT. Ameliorated by HRT
Mortality from heart disease	3% increase risk in women who do not take HRT
Dementia or neurocognitive dysfunction	Seen predominantly in women who do not take HRT
Parkinson's disease	Not significantly increased
Stroke	Not significantly increased

BC, breast cancer; HRT, hormone replacement therapy; OC, ovarian cancer; QoL, quality of life; RRSO, risk-reducing salpingo-oophorectomy.

[a]Two small studies in women undergoing RRSO do not demonstrate increase in risk of heart disease but these need to be interpreted with caution and should not be used to draw significant inferences.

 © 2021 Royal College of Obstetricians and Gynaecologists

14710528, 2022, 1, Downloaded from https://obgyn.onlinelibrary.wiley.com/doi/10.1111/1471-0528.16896 by University College London UCL Library Services, Wiley Online Library on [14/06/2023]. See the Terms and Conditions (https://onlinelibrary.wiley.com/terms-and-conditions) on Wiley Online Library for rules of use; OA articles are governed by the applicable Creative Commons License

Appendix IV: HRT adverse effects

**Estrogenic**	Breast tenderness<br>Fluid retention<br>Leg cramps<br>Nausea<br>Headaches
**Progestogenic**	Premenstrual syndrome-like symptoms<br>Nausea<br>Acne<br>Fluid retention<br>Bloating<br>Headache<br>Mood changes<br>Pelvic pain
**Androgen**	Hirsutism<br>Acne
**Other**	Erratic breakthrough uterine bleeding in first 3–6 months of continuous combined and long cycle HRT regimens

 © 2021 Royal College of Obstetricians and Gynaecologists

14710528, 2022, 1, Downloaded from https://obgyn.onlinelibrary.wiley.com/doi/10.1111/1471-0528.16896 by University College London UCL Library Services, Wiley Online Library on [14/06/2023]. See the Terms and Conditions (https://onlinelibrary.wiley.com/terms-and-conditions) on Wiley Online Library for rules of use; OA articles are governed by the applicable Creative Commons License

Appendix V: HRT and breast cancer risk following RRSO

Genetic risk factor	BC risk with HRT post RRSO	RRSO studies reporting HRT and BC risk[a]	Summary advice
BRCA1, *BRCA2*	No increase in primary risk if no personal history of BC	• BC with HRT post RRSO (HR 0.37, CI 0.14–0.96), similar to BC HR in overall RRSO cohort[49]<br>• *BRCA1* RRSO ever vs never HRT users (OR 0.58, CI 0.35–0.96; $P = 0.03$)[51]<br>• *BRCA1* RRSO ever versus never HRT users (OR 0.80, CI 0.55–1.16; $P = 0.24$)[50]<br>• *BRCA1* RRSO ever versus never HRT users (HR 0.97, CI 0.62–1.52; $P = 0.89$)[54]	HRT can be given up to age 51 if no personal history of BC and no other HRT contraindications.<br>Good prognostic TNBC: short-term HRT may be considered on a case-by-case basis.<br>ER+/PR+BC: No HRT

BC, breast cancer; ER+, estrogen receptor-positive; HR, hazard ratio; HRT, hormone replacement therapy; PR+, progesterone receptor-positive; RRSO, risk-reducing salpingo-oophorectomy; TNBC, triple negative breast cancer.
[a]These data are based on short-term outcomes. Additional well-designed studies with long-term outcomes are needed.

 © 2021 Royal College of Obstetricians and Gynaecologists

14710528, 2022, 1, Downloaded from https://obgyn.onlinelibrary.wiley.com/doi/10.1111/1471-0528.16896 by University College London UCL Library Services, Wiley Online Library on [14/06/2023]. See the Terms and Conditions (https://onlinelibrary.wiley.com/terms-and-conditions) on Wiley Online Library for rules of use; OA articles are governed by the applicable Creative Commons License

This Scientific Impact Paper was produced on behalf of the Royal College of Obstetricians and Gynaecologists by:

Professor R Manchanda MRCOG, London; Dr F Gaba MRCOG, London; Dr VS Talaulikar MRCOG, London; Dr J Pundir MRCOG, London; Dr S Gessler PhD, University College Hospital Gynaecological Cancer Research Centre; Miss MC Davies FRCOG, London; and Professor U Menon FRCOG, London.

The following individuals and organisations submitted comments at peer review:
Professor J Barwell, FRCP, University of Leicester; British Menopause Society; Mr DI Fraser FRCOG, Norwich; Professor F Guidozzi FRCOG, Johannesburg, South Africa; Dr M Hunter, PhD CPsychol AFBPS King's College London; UK Cancer Genetics Group; RCOG Women's Network; Dr WA Rocca MD, Mayo Clinic, Rochester, MN, USA; Dr AN Rosenthal FRCOG, UCL Hospitals NHS Foundation Trust, London; and Professor M Tischkowitz, PhD FRCP, University of Cambridge.

The Scientific Advisory Committee lead reviewer was: Dr N Potdar FRCOG, Leicester.

The Chair of the Scientific Advisory Committee was: Professor S Ghaem-Maghami[1] MRCOG, London; and Professor MD Kilby[2] FRCOG, Birmingham.
[1]until May 2018; [2]from June 2018

All RCOG guidance developers are asked to declare any conflicts of interest. A statement summarising any conflicts of interest for this Scientific Impact Paper is available from: www.rcog.org.uk/en/guidelines-research-services/guidelines/sip66.

The final version is the responsibility of the Scientific Advisory Committee of the RCOG.

The paper will be considered for update 3 years after publication, with an intermediate assessment of the need to update 2 years after publication.

DISCLAIMER

The Royal College of Obstetricians and Gynaecologists produces guidance as an educational aid to good clinical practice. They present recognised methods and techniques of clinical practice, based on published evidence, for consideration by obstetricians and gynaecologists and other relevant health professionals. The ultimate judgement regarding a particular clinical procedure or treatment plan must be made by the doctor or other attendant in the light of clinical data presented by the patient and the diagnostic and treatment options available.

 © 2021 Royal College of Obstetricians and Gynaecologists

14710528, 2022, 1, Downloaded from https://obgyn.onlinelibrary.wiley.com/doi/10.1111/1471-0528.16896 by University College London UCL Library Services, Wiley Online Library on [14/06/2023]. See the Terms and Conditions (https://onlinelibrary.wiley.com/terms-and-conditions) on Wiley Online Library for rules of use; OA articles are governed by the applicable Creative Commons License
